# Formative Structured Oral Examination as a Novel Method of Academic Mentoring for Slow and Medium Achievers in Medical Biochemistry: A Mixed-Method Study

**DOI:** 10.7759/cureus.104258

**Published:** 2026-02-25

**Authors:** Zachariah Bobby, Monisha Muralidharan, Hanumanthappa Nandeesha, Ramesh R, Devanatha Desikan V, Deepthi Sudha M, Zayapragassarazan Z

**Affiliations:** 1 Department of Biochemistry, Jawaharlal Institute of Postgraduate Medical Education and Research, Puduchery, IND; 2 Department of Biochemistry, Jawaharlal Institute of Postgraduate Medical Education and Research, Puducherry, IND; 3 Department of Medical Education, Jawaharlal Institute of Postgraduate Medical Education and Research, Puducherry, IND

**Keywords:** educational measurement, formative assessment, formative feedback, formative structured oral examination, slow and medium achievers, structured oral examination

## Abstract

Introduction: Academic mentoring is essential for graduate students who are slow or medium achievers. Yet, the usefulness of the Formative Structured Oral Examination (FSOE) as a tool for academic mentoring has not been studied. This study aims to assess the utility of FSOE as a tool for fostering academic mentoring among slow and medium achievers.

Methods: A convergent mixed-method study was conducted among 52 first-year MBBS students, covering three biochemistry topics with residents serving as resource persons. The study included both a quantitative single-group pre-post quasi-experimental component and an exploratory qualitative component (feedback and focus group discussions). Two cycles of FSOE were performed for each topic, with one student at a time and pre-prepared color cards. Each FSOE session lasted approximately one hour. Multiple-choice pre- and post-tests were conducted before and after the FSOE. Feedback was collected from students using a structured questionnaire employing a seven-point Likert scale. The questionnaire was reviewed by subject experts for content validity prior to administration, and focus group discussions were held among students and resource persons. Pre- and post-test scores were compared using a paired t-test for normally distributed variables and a Wilcoxon signed-rank test for non-normally distributed variables. P < 0.05 was considered significant. Analyses were done using IBM SPSS Statistics software, version 20 (IBM Corp., Armonk, NY, USA).

Results: FSOE implementation resulted in a significant improvement in post-test scores. Among low achievers, fat-soluble vitamin scores increased from 11 (8-13) to 17 (14-20) (p = 0.001; r = 0.85), while medium achievers also showed significant gains (p = 0.001; d = 1.21). Low achievers demonstrated significant improvement in amino acid metabolism and DNA replication (p < 0.05), with large effect sizes (d = 1.80 and 1.02). Scores further improved after the second FSOE cycle across all groups (p < 0.001). Compared with a topic taught without the FSOE, normalized pre- to post-test improvement showed large effect sizes across groups. Internal assessment scores improved significantly among low achievers (p < 0.005). Feedback and focus group discussions indicated enhanced conceptual understanding, improved engagement, reduced examination stress, increased motivation, and improvement in resource persons’ teaching skills, though logistical and inclusivity concerns were noted among the students.

Conclusions: The FSOE was an effective academic mentoring program that benefited both students and resource persons. The FSOE may be implemented as a regular mentoring program in medical biochemistry to address academic difficulties.

## Introduction

The teaching-learning process for slow and medium achievers remains a major challenge in medical education. In India, undergraduate medical education is mainly conducted through large-group didactic lectures. This method is largely teacher-centered and involves one-way communication, making it a passive form of learning. It plays a limited role in developing problem-solving skills and in improving the application of knowledge, particularly among slow and medium achievers. Therefore, there is a growing need to adopt more effective and learner-centered teaching strategies.

Currently, there are limited opportunities for teachers to identify the slow and medium achievers and to provide remedial measures for medical graduates. After identifying the slow and medium achievers, a one-to-one interaction is essential to promote their learning processes [1]. Some students find it challenging to understand the concepts of medical biochemistry and link them to their clinical significance. This leads them to rote learning and searching for the answers to essential questions without understanding the basic concepts [2]. Rote learning may lead to poor performance among some students.

In India, the graduate medical education (Bachelor of Medicine and Bachelor of Surgery (MBBS)) consists of three phases. Mentorship programs have been reported to enhance the academic performance of first-phase MBBS students [3]. However, limited information is available on academic mentoring among the first-phase medical students. To address this, we propose a novel approach to academic mentoring for underperforming students in medical biochemistry using the Formative Structured Oral Examination (FSOE) to support their academic performance.

The FSOE is designed to enhance the utility of oral exams by improving their validity and reliability when used as formative assessments [4-6]. In this method, questions are structured and developed sequentially from lower to higher levels of Bloom's taxonomy of cognition and are color-coded. Yet another advantage of the FSOE is that the keys and marks allotted to the questions are also provided to the examiner, thereby reducing examiner bias.

Formative assessments are assessments for learning, and the hallmark of formative assessment is the feedback given to learners, which promotes their learning [7]. Although formative assessments are commonly used in medical education, there is very little information on the use of the FSOE specifically for academic mentoring of undergraduate medical students in biochemistry. Therefore, this study was conducted to evaluate the usefulness of a modified FSOE as a mentoring tool to improve student learning.

## Materials and methods

Study design and participants

The study was conducted among first-phase graduate medical students at Jawaharlal Institute of Postgraduate Medical Education and Research, Puducherry, India, from February 2025 to July 2025. A convergent mixed-method study was conducted to evaluate the impact of FSOE on students and resource persons. The study included both a quantitative single-group pre-post quasi-experimental component and an exploratory qualitative component (feedback and focus group discussions). Quantitative methods were used to assess student performance through pretests, post-tests, and successive FSOE cycles (cycles 1 and 2). In addition, qualitative methods, such as feedback and focus group discussions, were used to explore learners' and resource persons' perceptions and experiences of this method. Using both quantitative and qualitative methods, the study explored both objective performance outcomes and their perceived learning experience. This convergent mixed-method study consisted of experiments on three topics: fat-soluble vitamins, amino acid metabolism, and DNA replication. The study adhered to the ethical guidelines outlined in the Indian Council of Medical Research (ICMR) guidelines for biomedical research involving human participants (2017) and the World Medical Association (WMA) Declaration of Helsinki (1964, revised), which governs medical research involving human participants [8,9]. The study was approved by the Institutional Ethical Committee of Jawaharlal Institute of Postgraduate Medical Education and Research, Puducherry, India (approval number: JIP/IEC-OS/2024/322). Informed consent was taken from all the participants.

At our institute, about 20% of students scored below 50% in the first two internal assessments, and another 16% scored between 50% and 60%. Thus, nearly 36% of students required academic mentoring.

Inclusion criteria

First-year MBBS students were grouped based on their previous internal assessment marks in biochemistry. Students scoring less than 50% in either the first or second internal assessments were considered slow achievers, and those scoring ≥50% but <60% in either assessment were considered medium achievers. Students from these groups who gave consent were included in the study.

Exclusion criteria

Unwilling participants were excluded from the study.

Sample size

As this was an exploratory educational intervention study, no formal sample size calculation was performed. Participation varied across the topics due to students' availability and academic scheduling, a limitation acknowledged. Overall, 52 students participated in the study, underwent the FSOE, and participated in pre- and post-test, which was 60% of the invited students.

Data collection and measurements

The FSOE was conducted for students after working hours, without affecting their regular teaching-learning programs, but before the subsequent internal assessments.

Structure of the FSOE

The FSOE consisted of pre-prepared color cards, which were designed to structure questioning across the cognitive levels, progressing from basic recall to higher-order reasoning. The color card was designed to enable a left-to-right progression of questions, with categories ranging from "must know" to "desirable to know" to "nice to know." From top to bottom, the questions were asked in sequence, covering recall, understanding, application, analysis, evaluation, and creativity, based on Bloom's taxonomy, and were scored according to the prefixed marks for each cognitive level. The answer keys and the maximum marks for the questions were provided on the color cards. Marks were allotted according to cognitive level and content category, using Bloom’s taxonomy. Each card included predefined questions, expected responses, and scoring criteria to ensure consistency and uniformity. The detailed scoring scheme and an example of a color card are provided in Appendix A.

Before the FSOE was conducted, the senior and junior residents of the department who were willing and available to participate underwent a briefing session to standardize the use of color cards and to train them in the questioning approach and scoring criteria. They served as resource persons for the conduct of the study. Although formal inter-rater reliability statistics were not calculated, the structured format and predefined scoring were intended to minimize the assessor variability. 

Two to three students were assigned to each resource person and worked on one topic at a time, such as fat-soluble vitamins, amino acid metabolism, or DNA replication. Prior to the FSOE intervention, students completed a 30-item multiple-choice questionnaire (MCQ) with a single best response (pre-test) for the respective topic (Appendix B).

During the FSOE session, questions were administered sequentially from lower- to higher-order cognitive levels according to Bloom’s taxonomy using color-coded cards. Resource persons conducted the FSOE individually with each student, either in person or online, based on feasibility. Each session lasted approximately one hour. Two FSOE cycles were conducted per student for the assigned topic to reinforce learning. When students were unable to answer, correct responses were explained and discussed during both cycles. Each question carried predetermined marks, and the total scores for cycles 1 and 2 were recorded in real time as students responded to the color card questions; these scores were subsequently compared.

After completion of the two FSOE cycles for that topic, a post-test was administered using the same set of MCQs to ensure comparability in content coverage and difficulty level.

Feedback

A seven-point Likert scale was employed to assess participants’ responses [10]. Each item was rated on a scale ranging from 1 to 7. For interpretative purposes, responses were categorized into three levels: scores of 1-2 were classified as “Not at all true,” indicating minimal or no agreement with the statement; scores of 3-5 were categorized as “Somewhat accurate,” reflecting a moderate level of agreement; and scores of 6-7 were classified as “Very true,” indicating strong agreement. This categorization facilitated meaningful interpretation of response patterns and enabled grouping of responses into low, moderate, and high levels for statistical analysis. The questionnaire was reviewed by subject experts for content validity prior to administration. The feedback questionnaire used in the study is provided in Appendix C. 

Focus group discussions

Volunteered students were invited to participate in the focus group discussion to examine the factors that facilitated or hindered their learning when the FSOE was used as a formative assessment tool. A separate focus group discussion was also held with the resource persons to further refine their approach. A total of two focus group discussions were conducted: one with participating students and one with the resource persons involved in the FSOE sessions.

A small group of 10 students was purposively selected for the focus group discussions to minimise deviation from the discussion points, which is more likely in larger groups. Each focus group discussion was guided by a set of 10 pre-developed, probing questions aligned with the study objectives. These questions explored perceptions of the FSOE process, perceived impact on learning, areas of improvement, feasibility, and overall acceptability. These questions were developed and validated by the investigators in alignment with the study objectives and evaluation framework. Each session lasted for one hour.

The discussions were moderated by a faculty member trained in medical education who was not directly involved in scoring the assessments, to minimize response bias. One of the investigators documented the discussion and subsequently transcribed the responses verbatim.

Data analysis

The Shapiro-Wilk test was used to assess normality. Data are presented as mean ± SD or median (IQR) based on the data distribution. Pre- and post-test scores were compared using the paired t-test or Wilcoxon signed-rank test, as appropriate. Fat-soluble vitamin scores (intervention component with FSOE) were normalized to water-soluble vitamin scores (non-intervention component) obtained from the same set of students to account for differences in overall academic performance. Normalization was performed by calculating the fat-soluble vitamin/water-soluble vitamin ratio separately for pre-test and post-test assessments and comparing them using the paired t-test. Feedback was summarised descriptively, and focus group discussions were analysed thematically. P <0.05 was considered statistically significant. Analyses were performed using IBM SPSS Statistics software, version 20 (IBM Corp., Armonk, NY, USA).

Effect sizes were calculated using Cohen’s d (d = t/√n) for paired t-tests and rank-biserial correlation (r = Z/√n) for Wilcoxon tests. 95% Confidence intervals (CIs) were calculated for all effect size estimates.

## Results

Comparison of pre-test and post-test scores across various topics

The pre-test and post-test scores are presented in Table 1. Post-test scores showed significant improvement following the implementation of the FSOE among slow and medium achievers, with a large effect size. 

**Table 1:** Comparison of Student Scores in the Pre-test and Post-test of the FSOE Data are presented as mean ± SD or median (IQR), as appropriate. Paired t-tests (t statistic) or Wilcoxon signed-rank tests (Z statistic) were used based on data distribution. Effect sizes are reported as Cohen’s d or rank-based effect size (r) with 95% confidence intervals. *P < 0.05 was considered statistically significant. FSOE: Formative Structured Oral Examination

Topic	Group	n	Pre-test (Max = 30 marks)	Post-test (Max = 30 marks)	Test statistic	p-value	Effect size (95% CI)
Fat-soluble vitamins	Slow achievers (Median (IQR))	15	11 (8–13)	17 (14–20)	−3.30	0.001*	0.85 (0.56–0.96)
	Medium achievers (Mean±SD)	13	11.00±3.83	15.92 ± 4.71	−4.38	0.001*	1.21 (0.47–1.95)
	Total students (Median (IQR))	28	11(8-13.75)	17 (13–20)	−4.38	<0.001*	0.83 (0.63–0.93)
Amino acid metabolism	Slow achievers (Mean±SD)	15	11.60±2.84	18.53 ± 2.77	−6.985	<0.001*	1.80 (0.98–2.62)
DNA replication		9	10.74±6.56	21.30 ± 4.23	−3.052	0.016*	1.02 (0.12–1.92)

Comparison of the FSOE cycle 1 and cycle 2 scores among the students

The scores from the FSOE cycle 1 and cycle 2 were assigned during the session as students responded to the colour card questions, thereby reflecting their performance in real time during the exercise.

Scores improved significantly after the second FSOE cycle across all groups and topics (p < 0.001). Both slow and medium achievers showed large effect sizes, indicating a strong positive impact of the FSOE on student performance as presented in Table 2.

**Table 2:** Comparison of Students’ Scores in Cycle 1 and Cycle 2 of the FSOE Data are presented as mean ± SD or median (IQR), as appropriate. Paired t-tests (t statistic) or Wilcoxon signed-rank tests (Z statistic) were used based on data distribution. Effect sizes are reported as Cohen’s d or rank-based effect size (r) with 95% confidence intervals. *P < 0.05 was considered statistically significant. FSOE: Formative Structured Oral Examination

Topic	Group	n	FSOE cycle 1 (Max =150 marks)	FSOE cycle 2 (Max =150 marks)	Test statistic	p-value	Effect size (95% CI)
Fat-soluble vitamins	Slow achievers (Median (IQR))	15	23.84 (16.89–44.70)	101.32 (94.37–123.18)	−3.40	0.001*	0.88 (0.30–1.46)
	Medium achievers (Mean±SD)	13	32.24 ± 14.51	112.17 ± 17.31	−12.28	<0.001*	3.41 (2.17–4.65)
	Total students (Median (IQR))	28	27.32 (17.01–44.70)	107.28 (95.12–124.67)	−4.62	<0.001*	0.87 (0.47–1.27)
Amino acid metabolism	Slow achievers (Mean±SD)	15	34.39 ± 11.70	101.14 ± 14.74	−16.32	<0.001*	4.22 (2.90–5.54)
DNA replication		9	42.89 ± 12.72	113.73 ± 14.38	−13.89	<0.001*	4.63 (2.27–6.99)

Comparison of normalized scores of the FSOE

Fat-soluble vitamin scores (intervention component with the FSOE) were normalized against water-soluble vitamin scores (non-intervention component) obtained from the same cohort of students to control for variations in overall academic performance. Normalization was carried out by calculating the fat-soluble vitamin/water-soluble vitamin ratio separately for the pre-test and post-test assessments, and the ratios were compared using a paired t-test, as shown in Table 3. The pre- to post-test improvement was associated with a large effect size among all the groups.

**Table 3:** Pre- and Post-test Scores of the FSOE on Fat-Soluble Vitamins (Test) Normalized With the Scores of Water-Soluble Vitamins (Control) Values are expressed as mean ± SD as appropriate. A paired t-test was applied based on the data distribution. Effect sizes are reported as Cohen’s d (paired t-test) with 95% confidence intervals. *P < 0.05 was considered statistically significant. FSOE: Formative Structured Oral Examination

Group	n	Normalised pre-test scores	Normalised post-test scores	Test statistics	p-value	Effect size (95% CI)
Slow achievers	15	0.93± 0.35	1.44± 0.36	7.237	<0.001*	1.87 (0.95 – 2.78)
Medium achievers	13	1.04± 0.25	1.56± 0.56	3.902	0.002*	1.08 (0.35 – 1.81)
Total	28	0.98± 0.31	1.50± 0.46	7.221	<0.001*	1.36 (0.82 – 1.90)

Comparison of marks of internal assessments conducted before and after the FSOE

Internal assessment marks before and after the FSOE intervention are presented in Table 4. The topic of fat-soluble vitamins among slow achievers showed a statistically significant improvement (41.40 ± 6.04 vs. 55.07 ± 16.80; t = −3.326, p = 0.005), with a large effect size (Cohen’s d = 0.86). In medium achievers, although mean scores increased after the FSOE (53.23 ± 1.48 vs. 58.46 ± 12.57), the difference was not statistically significant (t = −1.51, p = 0.156) and the effect size was small to moderate (Cohen’s d = 0.42). When all students were analysed together, a significant improvement in scores was noted after the FSOE (49 (41.25-53) vs. 56 (50.5-63); Z = −2.97, p = 0.003), with a moderate effect size (Cohen’s d = 0.56).

**Table 4:** Marks of Internal Assessments Before and After the FSOE Data are presented as mean ± SD or median (IQR), as appropriate. Paired t-tests (t statistic) or Wilcoxon signed-rank tests (Z statistic) were used based on data distribution. Effect sizes are reported as Cohen’s d or rank-based effect size (r) with 95% confidence intervals. *P < 0.05 was considered statistically significant. FSOE: Formative Structured Oral Examination

Group	Topic	n	Internal assessments before the FSOE (Max=100 marks)	Internal assessments after the FSOE (Max=100 marks)	Test statistics	p-value	Effect size (95% CI)
Slow achievers	Fat-soluble vitamins (Mean ± SD)	15	41.40±6.04	55.07±16.80	-3.326	0.005	0.86 (0.27–1.45)
Medium achievers	Fat-soluble vitamins (Mean ± SD)	13	53.23±1.48	58.46±12.57	-1.51	0.156	0.42 (−0.15–0.99)
Total	Fat-soluble vitamins (Median (IQR))	28	49 (41.25-53)	56 (50.5-63)	-2.97	0.003	0.56 (0.16–0.96)
Slow achievers	Amino acid metabolism and DNA replication (Median (IQR))	24	46 (41-48)	56(48-62)	-3.069	0.002	0.79 (0.21–1.37)

Similarly, among slow achievers, scores for amino acid metabolism and DNA replication increased significantly following the FSOE (46 (41-48) vs. 56 (48-62); Z = −3.069, p = 0.002), corresponding to a large effect size (Cohen’s d = 0.79).

However, topics beyond fat-soluble vitamins, amino acid metabolism, and DNA replication were also included in the internal assessment conducted after the FSOE. Therefore, improvement in the marks of internal assessment might not be exclusively due to the effect of the FSOE alone. 

Student feedback on the FSOE

Student feedback on the FSOE was analysed using Likert-scale responses and thematic analysis of free-text comments (Table 5 and Table 6). Key themes identified were improved conceptual understanding, reduced examination stress, increased motivation for self-directed learning, and inclusivity concerns. Interactive learning, peer-assisted discussions, and resource person mentorship facilitated engagement. While overall perceptions were positive, some variability in enjoyment and perceived effort was noted, and concerns regarding inclusivity were raised, suggesting that extending the intervention to all learners may enhance its acceptability.

**Table 5:** Student Feedback on the FSOE on Fat-Soluble Vitamins Collected Using a Likert Scale FSOE: Formative Structured Oral Examination

	Statement	Median and IQR (Likert scale range: 1–7)	Average response
A	I want this type of exercise in the future	6(4-7)	Very true
B	I experienced a stress-free environment of learning during the exercise	6(4.25-7)	Very true
C	I put a lot of effort into this activity	4.5(2.5-6)	Somewhat true
D	I feel this exercise is more beneficial for revision than self-study	5.5(3.25-7)	Somewhat true
E	This exercise has enhanced my self-directed learning process	5.5(4-7)	Somewhat true
F	I gained very much from this exercise	6 (4-7)	Very true
G	I enjoyed doing this activity very much	4 (4-6)	Somewhat true
H	The exercise inspired me towards learning	6 (4.25-6.75)	Very true

**Table 6:** Free Comments After the FSOE on Fat-Soluble Vitamins FSOE: Formative Structured Oral Examination

Verbatim student feedback	Code	Theme
“It was good. But it would be better to keep it for all students. Why bring partitions among us on the basis of marks!!”	Desire for inclusivity	Equity and inclusiveness in learning
“Good”	General positive perception	Overall acceptability of FSOE
“This learning idea will definitely improve our performances.”	Perceived academic benefit	Perceived improvement in academic performance
“It's a stress-free study method”	Reduced learning stress	Supportive and stress-free learning environment
“It’s so good”	High satisfaction	Learner satisfaction
“Thank you for helping us :)”	Appreciation for facilitation	Resource persons support and academic mentorship
“Understood how to learn biochem”	Improved learning strategy	Development of effective learning strategies
“I want an interactive session with JR. It was very good, and I learned so much from them”	Value of peer–teacher interaction	Interactive and peer-assisted learning
“Yes, it was a better way of revising a topic peripherally and motivating us to perform well in internals with more self-confidence than before.”	Effective revision and confidence-building	Enhanced revision, motivation, and self-confidence
“It's okay, we can have this at least, we will know there's a lot to read and what is important to read first.”	Content prioritization	Guidance in focused and strategic learning
“Spl Thanks to our agar sir. 🙏🏻🙌🏻 Big fan.”	Faculty inspiration	Positive faculty influence
“It is very helpful”	Utility of intervention	Perceived usefulness of FSOE
“I wish to write more tests, so that I can check how much I’ve retained”	Desire for formative assessment	Motivation for self-assessment and retention monitoring

Focus group discussions

We conducted focus group discussions with students and resource persons separately to discuss the merits and demerits of FSOE.

Thematic Analysis of Focus Group Discussion With Students

The questions asked during the session and the responses of the students are presented in Table 7. The analysis of student responses revealed four key themes: acceptability and engagement, enhanced learning, immediate feedback and structured learning, and logistical challenges.

**Table 7:** Responses of Students During the Focus Group Discussion FSOE: Formative Structured Oral Examination

Questions asked during the focused discussion	Summary of responses
1. Did you like and enjoy your present experience with the FSOE? Or was it a boring exercise for you?	The students liked the exercise.
2. Do you feel the time you spent with us for the FSOE was more beneficial than if you were to spend the same time on self-study? Why?	The time spent on the FSOE was more beneficial than self-study, as it provided feedback and clarified doubts.
3. What are the advantages of this exercise when compared to self-study in promoting your learning process?	Compared to self-study, the FSOE takes less time, helps identify lacunae, and provides focused attention on critical areas.
4. What elements in the FSOE promoted your learning process of the given topic?	The FSOE helped to complete a topic within a set deadline, and answers could be obtained without much delay, making the exercise student-friendly
5. Compare and comment on your attention span during the FSOE with self-study. How engaging was the FSOE for you?	More engaging than self-study, with fewer distractions, and a better attention span than a lecture class.
6. Did the FSOE promote your learning of the depth of the topic, problem-solving abilities, and reasoning skills? If so, please explain. Intellectually, how challenging was the exercise for you?	Helped to understand the concepts in a better way, improved reasoning skills, and helped to connect topics
7. What are the limitations of this exercise in comparison to self-study? Did you find it challenging to participate in this exercise? If so, please explain	Students were tired after working hours, due to inconvenient timing and practical difficulties in attending physical classes, with some students preferring the offline mode and others the online mode. It was suggested that sessions be scheduled on Saturdays or Sundays.
8. Did the present exercise address any of your difficulties in learning biochemistry?	The doubts were clarified on the spot, and concepts were understood better.
9. Did this exercise bring about any significant changes in your approach to learning Biochemistry?	As one topic was studied in depth, it became a more interesting subject.
10. Do you have any suggestions for educators or learners who want to conduct and participate in the FSOE more effectively in their biochemistry studies?	It was suggested that the FSOE be conducted immediately after the regular lecture class for more effective learning.

Acceptability and engagement: Students found the FSOE enjoyable and more engaging than self-study or traditional lectures, reporting better attention span and fewer distractions.

Enhanced learning: Participants noted improved conceptual understanding, reasoning skills, and integration of topics. The focused format helped identify learning gaps and promoted in-depth study.

Immediate feedback and structured learning: Immediate clarification of doubts and guided questioning were perceived as major strengths. The structured, time-bound format was considered student-friendly and more beneficial than self-study.

Logistical challenges: Fatigue after working hours, inconvenient timing, and preferences for online versus offline sessions were reported. Students suggested scheduling sessions immediately after regular lectures or on weekends to improve participation.

Thematic Analysis of Resource Persons’ Focus Group Discussion

The questions asked and responses of the resource persons during the focus group discussion are presented in Table 8. Five key themes emerged from the analysis: student engagement, professional development, structured learning benefits, limitations, and suggestions for improvement.

**Table 8:** Responses of Resource Persons During the Focus Group Discussion FSOE: Formative Structured Oral Examination

Questions asked during the focused discussion	Summary of responses
1. Did you like and enjoy your present experience with the FSOE? Or was it a boring exercise for you?	- Highly motivated students made the session smooth, but less motivated students were hesitant, scared, or apprehensive, reducing interaction and making the process less engaging. - With a large number of students, the same questions and explanations had to be repeated multiple times, creating monotony for the resource person. - Even after repeated prompts, some students gave little or no response, which was discouraging for the resource person - Conducting after the regular class hours made students more apprehensive and less responsive, further reducing the engagement.
2. Was this a learning opportunity for you in Biochemistry and in teaching skills when you conducted the FSOE? If so, please explain.	- All resource persons agreed that conducting the FSOE was a valuable learning opportunity, particularly for enhancing teaching skills. - The one-to-one interaction allowed them to understand each student’s level of knowledge and areas of difficulty more effectively. This format also enabled on-the-spot improvement in teaching approaches, such as using relevant images and visual aids to clarify concepts during the session.
3. Do you think this exercise is more advantageous compared to self-study in promoting the learning process of the students you handled? If so, what are the advantages?	Depends on the student’s learning style and motivation.
4. What elements in the FSOE do you think would have promoted the learning process of the students in the given topic?	- Starting with basic concepts and gradually progressing to higher-order thinking helped students understand the topic more effectively. - The stepwise approach encouraged logical building of concepts and better retention. - Focusing on the subsections of a single topic allowed for deep understanding. - The one-to-one interaction provided a comfortable environment for students to ask questions freely. - Students could clarify doubts immediately, leading to better comprehension.
5. What are the limitations of this exercise in comparison to self-study?	- Timing constraints – Finding a mutually convenient time for both the student and the resource person was challenging sometimes. - The exercise was restricted to only a few topics.
6. Did you find it challenging to conduct this exercise? If so, please explain.	- Depends on how cooperative the students are. - Hard to conduct before IA exams as students prefer to study on their own.
7. Do you have any suggestions for educators or learners who want to conduct and participate in FSOE more effectively in their biochemistry studies?	Allow students to choose the topic, so they are more interested and engaged.
8. Was the exercise educationally engaging for the resource persons and the students?	Yes, the exercise was engaging, promoting active learning for students and enabling resource persons to guide them effectively.
9. Did you plan and prepare specifically for the exercise? How did this exercise help you to think differently in learning biochemistry?	Yes, the resource persons conducted planning and preparation for the exercise. It helped to think more critically and practically apply biochemistry concepts.
10. Were you able to assess the students well during the learning session?	Yes, students were well assessed since it was a one-to-one session.

Student engagement: Sessions were smooth with motivated students, but less interactive with hesitant or less responsive learners. Repetition of content and post-class timing sometimes reduced engagement.

Professional development: Resource persons viewed the FSOE as a valuable opportunity to enhance teaching skills. The one-to-one format helped identify students’ knowledge gaps and allowed adaptive teaching using visual aids and real-time clarification.

Structured learning benefits: A stepwise progression from basic to higher-order concepts promoted logical understanding and retention. Focused discussion of single topics and immediate doubt clarification enhanced comprehension.

Limitations: Scheduling difficulties, restriction to limited topics, student cooperation, and timing near internal assessments were noted as challenges.

Suggestions: Allowing students to choose topics and scheduling sessions more conveniently were recommended. Overall, the FSOE was considered educationally engaging and effective for student assessment.

## Discussion

There are several debates on the utility of the FSOE as a tool for summative assessments [10-14]. However, limited information is available in the literature regarding the utility of the FSOE [15]. Shenoy et al. obtained perspectives from fellows and faculty on the educational effectiveness, feasibility, and acceptability of the FSOE for formative feedback. They found that the FSOE utilised for formative feedback was perceived by fellows and faculty to have several educational advantages, with high acceptability [16]. We conducted a mixed-method study to evaluate the utility of the FSOE for academic mentoring of first-year MBBS students in medical biochemistry who are slow or medium achievers at our institute.

Both the quantitative and qualitative analyses reflected the utility of the FSOE as a tool for fostering academic mentorship among first-year MBBS students. There were significant increases in post-test scores and oral examination scores after the second cycle. The feedback and free comments from students, as well as the responses from the focus group discussion with students and resource persons, supported the usefulness of FSOE in addressing the learning difficulties of slow and medium achievers in biochemistry.

The FSOE has several advantages for enhancing students' learning. In general, assessments are evaluated based on their validity, reliability, feasibility, acceptability, and educational impact. Unlike in summative assessments, reliability is of less significance in formative assessments. The objective of formative assessments is to promote learning, whereas the purpose of summative assessments is decision-making. The major criticism of oral examinations is their subjectivity, which renders them unreliable. However, the subjectivity of oral examinations is a blessing in disguise, as it contributes to improving assessment validity. The flexibility possible in conducting viva voce generally enables the assessor to frame questions tailored to the needs of the candidates [17]. This is especially significant when assessing the requirements for students to provide feedback. The hallmark of formative assessment is feedback, which thereby promotes student learning. Therefore, the subjectivity of the FSOE offers a greater opportunity for student learning [16]. The interactions between the student and assessor might vary, which provides a better opportunity to identify student problems and correct them. In the viva voce, the depth of knowledge and clinical reasoning abilities are better tested in comparison to written exams [12]. Khilnani et al. emphasize that oral examination can provide an opportunity to “evaluate the depth of knowledge, application, and analytical capability, ethics, and professionalism” [12].

The FSOE has the advantage of requiring little preparation and execution time for the assessors since the questions, answers, and allotted marks are already available in the color card template. In fact, in the color card system that we created, there is a two-dimensional progression. From left to right, questions are asked in the sequence of must know, desirable to know, and nice to know. From top to bottom, questions are framed in increasing order of Bloom's taxonomy of cognition. This design of the color card enables resource persons to provide feedback sequentially as the candidate answers.

In traditional viva theory, a discussion in the form of questions and answers takes place between the examiner and candidate, in the absence of a patient [3,4]. In contrast to traditional viva or oral examinations, the FSOE uses blueprints/checklists to structure the questions/discussion and requires examiners to adequately document answers and scores to provide feedback [5]. While it appears to perform better on the psychometric plan than traditional/unstructured oral examination [5,6], the FSOE still presents reliability issues related to content specificity and inter-rater variance [6,11-12].

The qualitative analyses illustrate the usefulness of the FSOE as a tool for academic mentoring exercises, as well as its few limitations. As for the students, they gained a great deal from this exercise, and the entire process inspired them to learn. The FSOE helped them understand the concepts more straightforwardly, improved their reasoning skills, and facilitated connections between topics. Compared to self-study, the FSOE takes less time, helps identify areas for improvement among students, and provides them with an opportunity to focus on key areas.

The resource persons enjoyed sessions with highly motivated students. However, with less motivated students, the process was less engaging, as they were hesitant, scared, or apprehensive, leading to fewer interactions. In general, the resource persons felt that conducting the FSOE was a valuable learning opportunity, particularly for enhancing teaching skills. The one-to-one interaction allowed them to better understand each student’s level of knowledge and areas of difficulty. This format also enabled on-the-spot improvement in teaching approaches, such as using relevant images and visual aids to clarify concepts during the session. Starting with basic concepts and gradually progressing to higher-order thinking helped students better understand the topics.

Educational impact refers to the effect of any intervention, such as an assessment, on the learning process [18]. The Kirkpatrick hierarchy of program evaluation can assess the educational impact of assessment [19]. These include learners’ reactions, modifications of attitudes, and the actual learning that occurs at the lowest levels of educational impact. From the quantitative and qualitative data obtained, it is evident that all stakeholders perceived or experienced positive academic effects of the FSOE on student learning.

One major limitation of the FSOE was student fatigue from sessions held after working hours, leading some students to prefer the online mode. Scheduling difficulties for in-person sessions were noted, and students suggested weekend sessions as a possible solution. Focus group discussions also suggested allowing students to choose topics to improve engagement and reduce concerns about segregating slow and medium achievers. In addition, preparing color cards was time-consuming for teachers. The absence of a systematic qualitative analytical framework limits the interpretative strength of the study findings. This is acknowledged as a limitation. Selection, response, and recall bias could not be excluded due to reduced participation of medium achievers in later topics, mixed online and offline modes, and the use of identical MCQs.

## Conclusions

The FSOE emerged as a structured, learner-centred mentoring strategy that improved academic performance, learner engagement, and faculty teaching skills. Its feasibility and acceptability suggest potential for integration into routine formative assessment in medical biochemistry. Future studies may use larger samples and include all achievement categories.

## Figures and Tables

**Figure 1:**
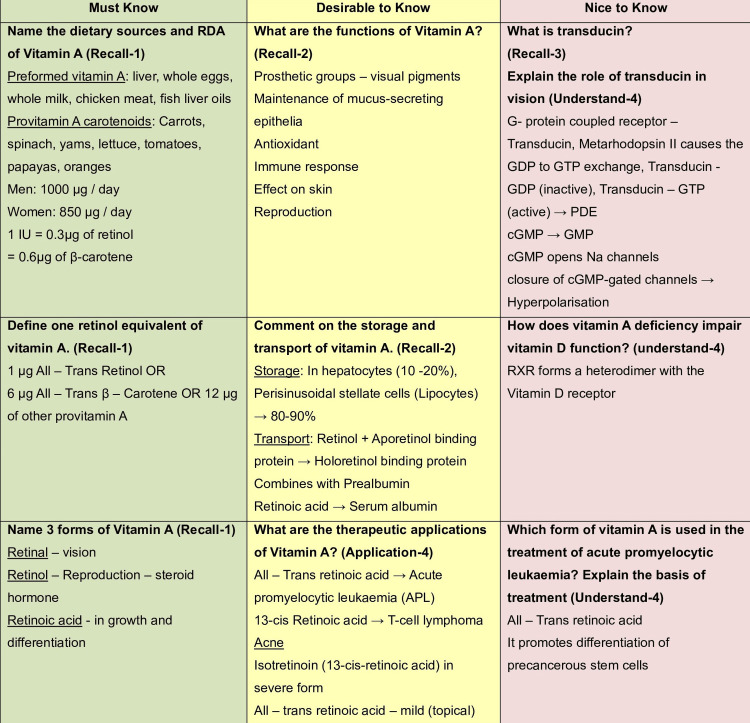
Model colour card of fat-soluble vitamins with scoring Marks are allotted based on cognition level and content category: Must Know: Recall – 1 mark, understand – 2 marks, apply – 3 marks, Analyse – 4 marks, evaluate – 5 marks, Create – 6 marks Desirable to Know: Recall – 2 marks, understand – 3 marks, apply – 4 marks, Analyse – 5 marks, evaluate – 6 marks, Create – 7 marks Nice to Know: Recall – 3 marks, understand – 4 marks, apply – 5 marks, Analyse – 6 marks, evaluate – 7 marks, Create – 8 marks

**Figure 2:**
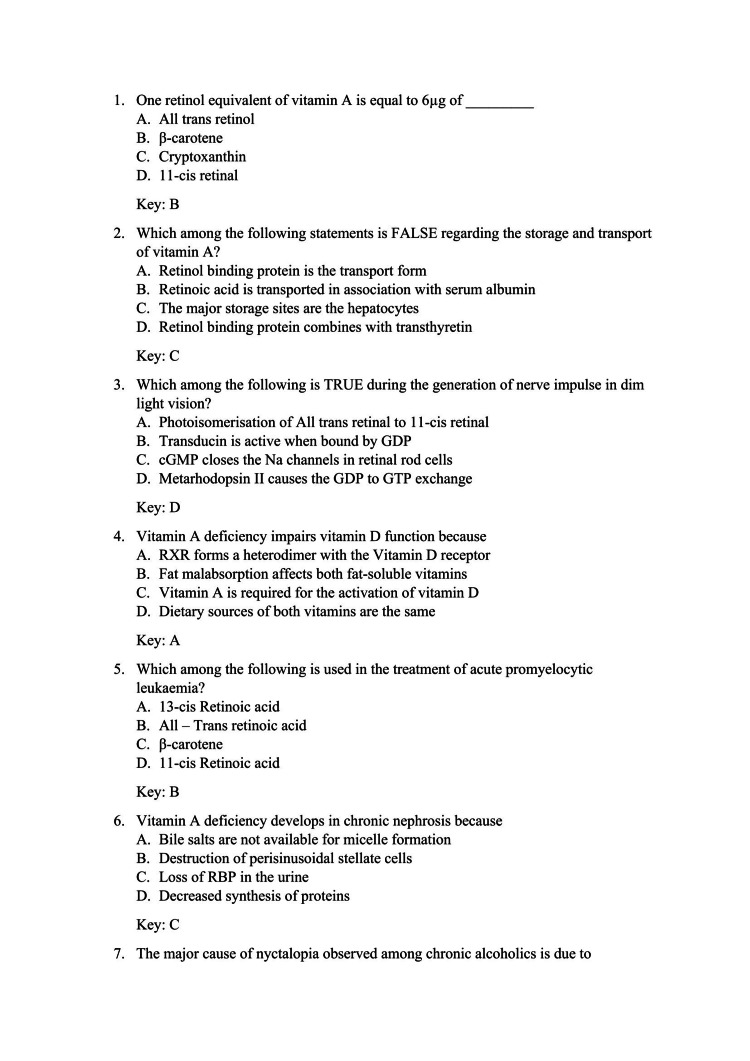
Fat-soluble vitamins multiple-choice questionnaire: part 1

**Figure 3:**
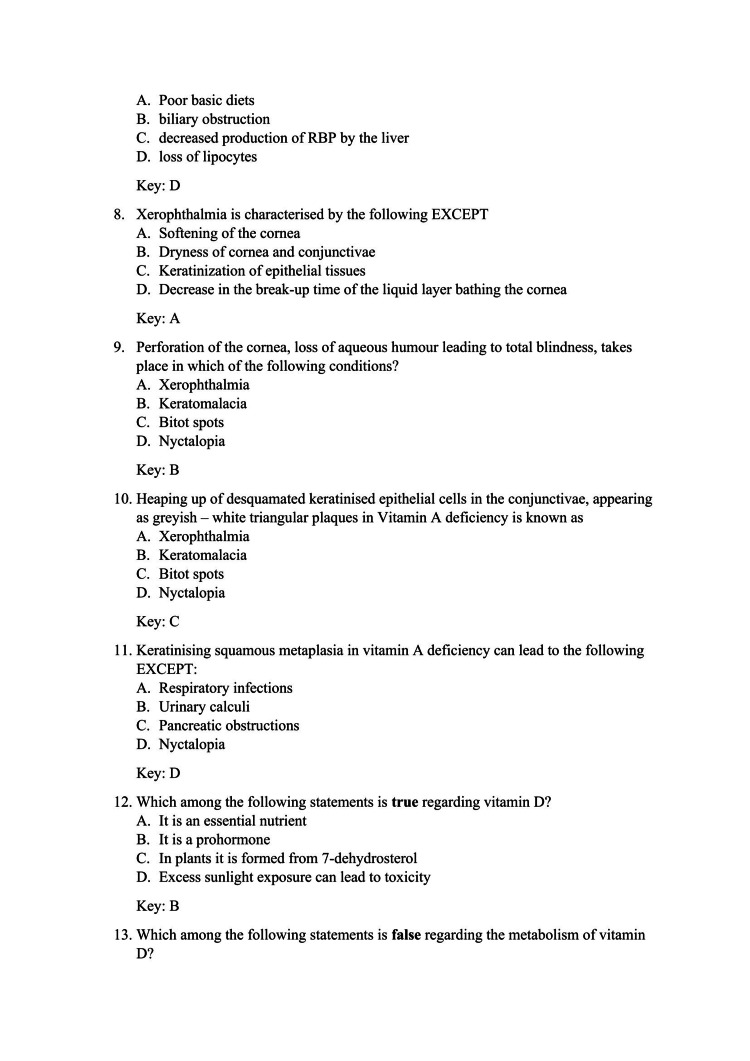
Fat-soluble vitamins multiple-choice questionnaire: part 2

**Figure 4:**
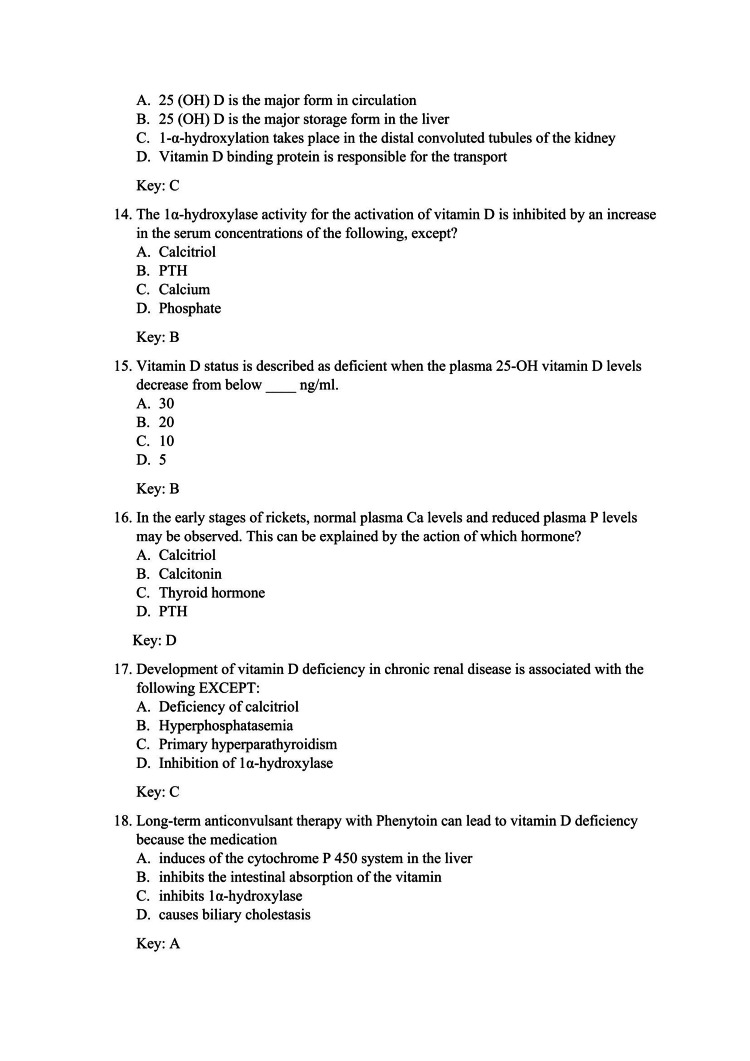
Fat-soluble vitamins multiple-choice questionnaire: part 3

**Figure 5:**
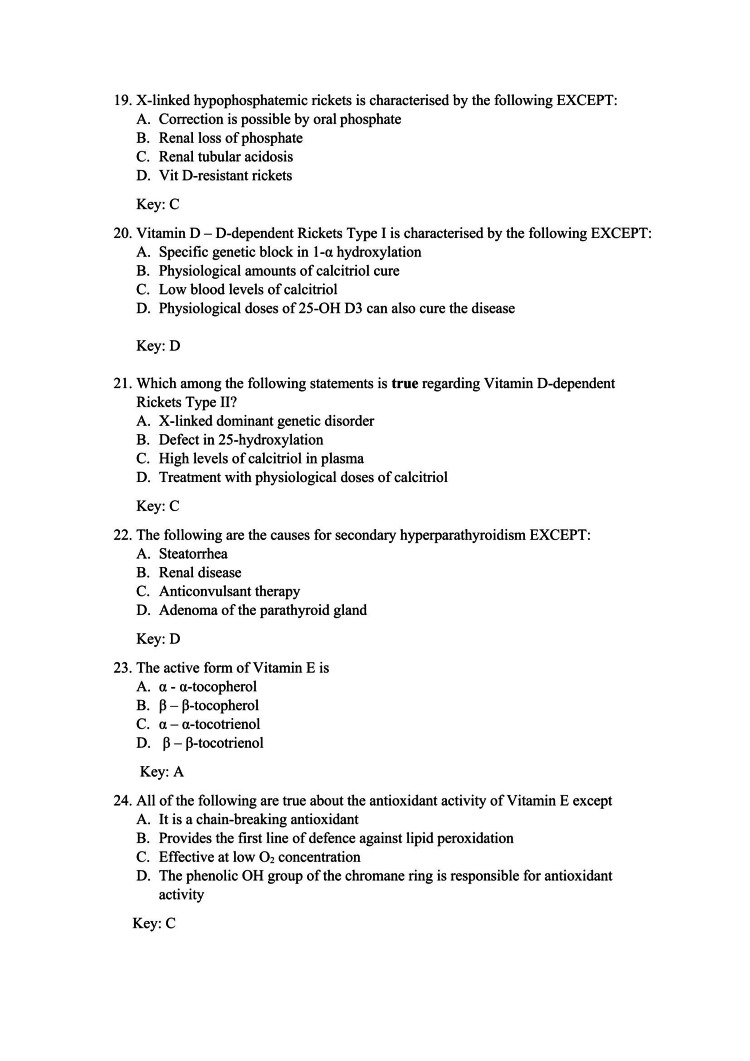
Fat-soluble vitamins multiple-choice questionnaire: part 4

**Figure 6:**
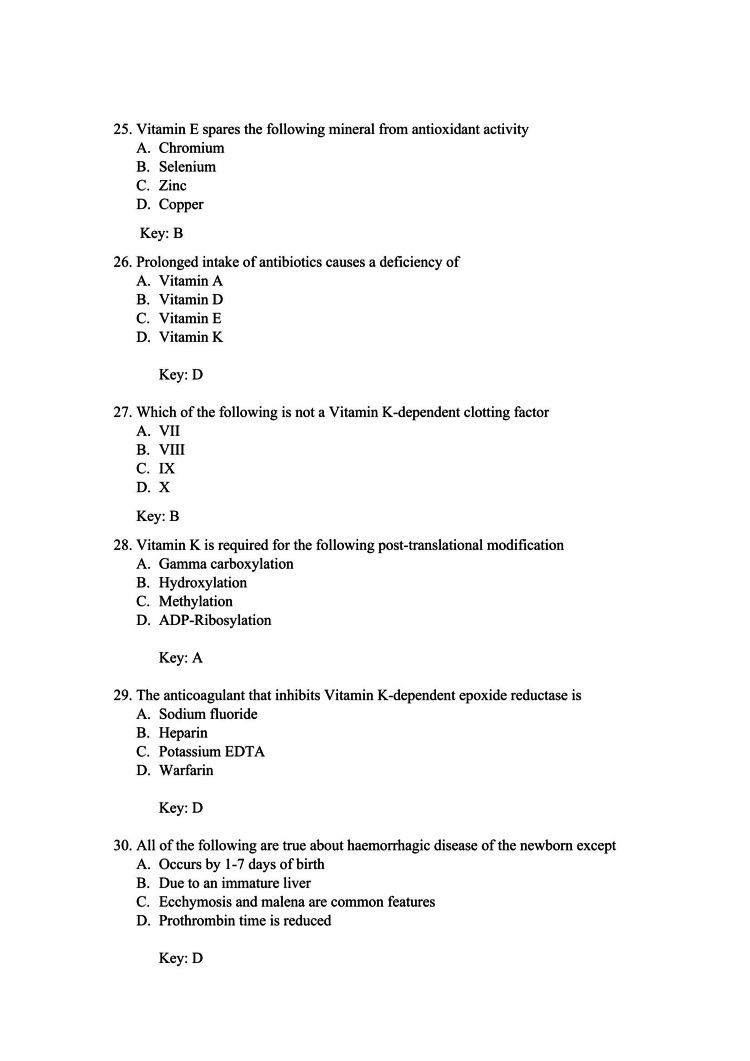
Fat-soluble vitamins multiple-choice questionnaire: part 5

**Figure 7:**
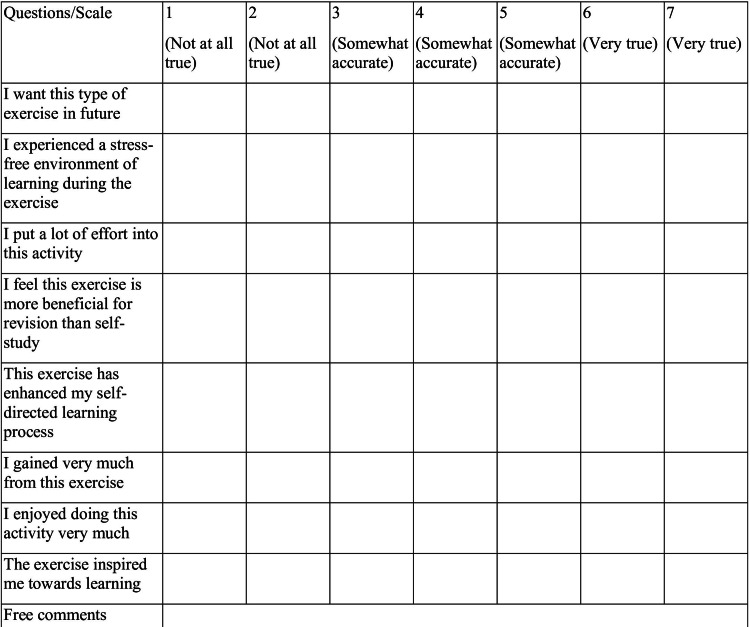
Feedback questionnaire The scale: 1 and 2 (Not at all true), 3, 4, and 5 (Somewhat accurate), and 6 and 7 (Very true).

## Appendices

Appendix A 

Appendix B 

Appendix C 

## References

[1] Banna J, Grace Lin MF, Stewart M, Fialkowski MK (2015). Interaction matters: strategies to promote engaged learning in an online introductory nutrition course. J Online Learn Teach.

[2] Spicer DB, Thompson KH, Tong MS, Cowan TM, Fulton TB, Lindsley JE (2019). Medical biochemistry without rote memorization: multi-institution implementation and student perceptions of a nationally standardized metabolic map for learning and assessment. Med Sci Educ.

[3] Guhan N, Krishnan P, Dharshini P, Abraham P, Thomas S (2020). The effect of mentorship program in enhancing the academic performance of first MBBS students. J Adv Med Educ Prof.

[4] Shenwai MR, B Patil K (2013). Introduction of structured oral examination as a novel assessment tool to first year medical students in physiology. J Clin Diagn Res.

[5] Loomba R, Jindal NM (2024). Improving process aspect of oral examination as assessment tool in undergraduate biochemistry by introducing structured oral examination: an observational study in India based on a survey among stakeholders. Korean J Med Educ.

[6] Abuzied AI, Nabag WO (2023). Structured viva validity, reliability, and acceptability as an assessment tool in health professions education: a systematic review and meta-analysis. BMC Med Educ.

[7] Morris R, Perry T, Wardle L (2021). Formative assessment and feedback for learning in higher education: a systematic review. Review Educ.

[8] World Medical Association (2013). World Medical Association Declaration of Helsinki: ethical principles for medical research involving human subjects. JAMA.

[9] (2017). National ethical guidelines for biomedical and health research involving human participants. https://ethics.ncdirindia.org/asset/pdf/ICMR_National_Ethical_Guidelines.pdf.

[10] Bobby Z, Desikan VD, Zayapragassarazan Z (2025). Concept mapping as a tool for fostering self-directed learning among graduate medical students in biochemistry: an experimental, analytical study. Biochem Mol Biol Educ.

[11] Puppalwar P, Rawekar A, Chalak A, Dhok A, Khapre M (2014). Introduction of objectively structured viva-voce in formative assessment of medical and dental undergraduates in biochemistry. J Res Med Educ Ethics.

[12] Khilnani AK, Charan J, Thaddanee R, Pathak RR, Makwana S, Khilnani G (2015). Structured oral examination in pharmacology for undergraduate medical students: factors influencing its implementation. Indian J Pharmacol.

[13] Jefferies A, Simmons B, Ng E, Skidmore M (2011). Assessment of multiple physician competencies in postgraduate training: utility of the structured oral examination. Adv Health Sci Educ Theory Pract.

[14] Rasalkar K, Tripathy S, Sinha S (2025). Enhancing medical assessment strategies: a comparative study between structured, traditional and hybrid viva-voce assessment. BMC Med Educ.

[15] Boulais I, Ouellet K, Lachiver EV, Marceau M, Bergeron L, Bernier F, St-Onge C (2023). Considering the structured oral examinations beyond its psychometrics properties. Med Sci Educ.

[16] Shenoy RV, Newbern D, Cooke DW (2022). The structured oral examination: a method to improve formative assessment of Fellows in pediatric endocrinology. Acad Pediatr.

[17] Davis MH, Karunathilake I (2005). The place of the oral examination in today's assessment systems. Med Teach.

[18] van der Vleuten CP, Schuwirth LW (2005). Assessing professional competence: from methods to programmes. Med Educ.

[19] Rouse DN (2011). Employing Kirkpatrick's evaluation framework to determine the effectiveness of health information management courses and programs. Perspect Health Inf Manag.

